# Terlipressin vs Midodrine Plus Octreotide for Hepatorenal Syndrome-Acute Kidney Injury: A Propensity Score–Matched Comparison

**DOI:** 10.14309/ctg.0000000000000627

**Published:** 2023-08-25

**Authors:** Stevan A. Gonzalez, Viktor V. Chirikov, Wei-Jhih Wang, Xingyue Huang, Khurram Jamil, Douglas A. Simonetto

**Affiliations:** 1 Division of Hepatology, Annette C. and Harold C. Simmons Transplant Institute, Baylor Scott & White All Saints Medical Center, Fort Worth, Texas, USA;; 2 Department of Medicine, Burnett School of Medicine at TCU, Fort Worth, Texas, USA;; 3 OPEN Health, Parsippany, New Jersey, USA;; 4 Mallinckrodt Pharmaceuticals, Hampton, New Jersey, USA;; 5 Division of Gastroenterology and Hepatology, Mayo Clinic College of Medicine and Science, Rochester, Minnesota, USA.

**Keywords:** real-world data, retrospective chart review, vasopressin analog, midodrine, octreotide

## Abstract

**INTRODUCTION:**

Evidence on the comparison of treatments for hepatorenal syndrome-acute kidney injury (HRS-AKI) in a US population is limited. An indirect comparison of terlipressin plus albumin vs midodrine and octreotide plus albumin (MO) may provide further insight into treatment efficacy.

**METHODS:**

Cohorts of patients treated for HRS-AKI characterized by inclusion of patients with serum creatinine (SCr) <5 mg/dL and baseline acute-on-chronic liver failure grades 0–2 and exclusion of patients listed for transplant if model for end-stage liver disease scores ≥35 were pooled from (i) the CONFIRM and REVERSE randomized controlled trials (N = 159 meeting eligibility criteria from N = 216 overall, treated with terlipressin) and (ii) a retrospective review of medical records from 10 US tertiary hospitals (2016–2019; N = 55 treated with MO meeting eligibility criteria from N = 200 overall). The primary end point comparing the 2 cohorts was HRS reversal defined as achieving SCr ≤1.5 mg/dL at least once during the treatment. Covariate balancing propensity scoring was used to adjust for differences in baseline characteristics.

**RESULTS:**

HRS-AKI reversal was achieved in 52.35% of terlipressin-treated patients compared with 20% of MO-treated patients (adjusted mean difference 32.35%, 95% confidence interval [CI] 17.40–47.30, *P* < 0.0001). Terlipressin-treated patients had increased overall survival (adjusted hazard ratio 0.57, 95% CI 0.35–0.93, *P* = 0.02) but similar transplant-free survival (adjusted hazard ratio 0.79, 95% CI 0.53–1.17, *P* = 0.24). Achievement of HRS-AKI reversal was associated with increased OS and TFS regardless of treatment (*P* < 0.001).

**DISCUSSION:**

Consistent with prior reports, terlipressin plus albumin is more effective in improving kidney function and achieving HRS-AKI reversal than MO plus albumin based on indirect comparison in a US population.

## INTRODUCTION

Hepatorenal syndrome-acute kidney injury (HRS-AKI) is a late complication of cirrhosis characterized by rapid deterioration in kidney function among patients with cirrhosis, resulting from reduced kidney perfusion in the setting of portal hypertension and renal vasoconstriction (1,2). HRS marks a milestone in the progression of cirrhosis, with associated high mortality after onset (2–5). More than one-third of patients with HRS-AKI do not survive to discharge, and among those who survive, one-third are readmitted to the hospital within 30 days (6).

Treatment guidelines on HRS-AKI in the United States recommend the use of albumin plus a vasoconstrictor for up to 14 days until serum creatinine (SCr) levels decrease to ≤1.5 mg/dL or within 0.3 mg/dL of baseline (2). The current standard of care in the United States has included albumin with unlabeled use of midodrine plus octreotide (MO) often as first-line therapy, while albumin with norepinephrine is reserved for use in an intensive care unit (ICU) setting through a central line and continuous monitoring. By contrast, terlipressin, an analog of vasopressin that has been approved in Europe for many years (7), is recommended as the vasoconstrictor of choice over other agents such MO or norepinephrine (8) and has recently been approved for HRS-AKI by the US Food and Drug Administration (FDA).

No published data are currently available comparing HRS-AKI treatments recommended for use in a US population. A recent retrospective review conducted on the treatment of HRS-AKI in US hospitals identified patients who benefit the most from vasoconstrictor therapy, which included those presenting with SCr <5 mg/dL and baseline acute-on-chronic liver failure (ACLF) grades 0–2 and excluded those with model for end-stage liver disease (MELD) scores ≥35 if listed for transplant, with most patients treated with MO (9).

Therefore, the objective of this study was to conduct an indirect treatment comparison of terlipressin with MO among patients with HRS-AKI with serum creatine <5 mg/dL and baseline ACLF grades 0–2 and excluding those with MELD scores ≥35 if listed for transplant, consistent with the expected and recommended use of terlipressin in HRS-AKI. Patient-level data on terlipressin from the CONFIRM (NCT02770716) and REVERSE (NCT01143246) trials, which enrolled patients diagnosed with type 1 HRS-1 (HRS-AKI) and excluding those with sepsis and/or uncontrolled bacterial infection or severe cardiovascular disease, were pooled and compared with patient-level real-world data of patients with HRS-AKI identified from a US chart review.

## METHODS

In this study, individual-level patient data were available for terlipressin plus albumin from pooled clinical trial data and on MO from a US retrospective chart review. To overcome the challenge of limited head-to-head comparisons, a matching approach was implemented to indirectly compare the effectiveness of terlipressin with standard-of-care regimens (10–12). Several steps were performed to: (i) define the study cohorts and identify relevant treatment comparators from the US chart review; (ii) select and adjust key factors to balance patient characteristics at baseline; and (iii) compare unadjusted and adjusted health outcomes between comparators. The retrospective chart review of the study was approved by the Western institutional review board/Copernicus Group institutional review board, which granted a waiver for obtaining informed consent from patients.

### Data source

#### Trial data on terlipressin

Patient-level data on the terlipressin cohorts from the CONFIRM and REVERSE trials were used in this study. While the OT-0401 trial was also considered for inclusion, it was not included because it did not contain baseline information on whether patients were listed for transplant, which was one of the criteria to define our HRS-AKI patient study population.

#### Real-world treatment data from US chart review

Retrospective medical record review data included a total of 200 adult patients presenting with HRS-AKI at 10 tertiary care centers between January 1, 2016, and June 30, 2019. Participating investigators (or their designees) identified patients with a clinical diagnosis of HRS-AKI based on expert review and collected data from hospital admission to 90 days postdischarge or until death using an electronic case report form hosted on a secure website. Tertiary centers were experienced in diagnosis of HRS, both as part of their clinical practice and participants in clinical trials in HRS with strict eligibility criteria; 6 of the 10 tertiary care centers and their principal investigators were also participating sites for the CONFIRM trial.

Most patients in the US chart review were treated with MO plus concomitant albumin and therefore comprised the main comparator cohort to the terlipressin plus albumin cohorts from the clinical trials. Patients were included in the MO cohort if they used MO as the initial treatment and received the treatment for at least 2 days. In addition, midodrine and octreotide had to be used within 2 days of each other to be considered an MO combination. Patients in the MO cohort who switched to norepinephrine as subsequent treatment and used it for more than 1 day were excluded from the MO cohort.

### Study population

Chart review patients were identified using International Classification of Disease, 9th revision (*ICD-9*) code of 572.4 and *ICD-10* code of K76.7 or N17.9 (acute renal failure, unspecified) with K74.6 (other and unspecified cirrhosis of the liver) and/or based on the documentation of HRS-AKI diagnosis (subject to individual investigator's determination, including capture of primary reason for hospital admission, International Club of Ascites [ICA] HRS criteria met, setting of diagnosis, and precipitating events) within patients' medical charts. Overall, 30.3% of the patients met all 6 ICA-HRS criteria at diagnosis, 49.5% met at least 5 ICA-HRS criteria, and 73.7% met at least 4 criteria at diagnosis. Verification of every ICA HRS-AKI criterion component was limited by the retrospective design of this study. The most commonly met ICA-HRS criteria were presence of cirrhosis and ascites (94%), followed by SCr >1.5 mg/dL (86.4%). Overall, 36.4% of patients were reported to have an *ICD-9*/*ICD-10* code for HRS-AKI diagnosis at their first hospitalization, while the remaining 63.6% patients were identified solely based on physician diagnosis.

Terlipressin clinical trials defined patients with HRS-AKI as rapidly progressive worsening in kidney function to a SCr level at least 2.25 mg/dL and meeting a trajectory for SCr to double over 2 weeks and no sustained improvement in kidney function (less than 20% decrease in SCr and SCr at least 2.25 mg/dL) at least 48 hours after diuretic withdrawal and the beginning of plasma volume expansion with albumin.

To create a more comparable cohort for the purposes of this study, patients were excluded if they met any of the following: had no data on treatment and clinical outcomes; had missing baseline values for SCr, bilirubin, international normalized ratio, ACLF or MELD score, or had baseline SCr <1.5 mg/dL. Patient SCr values were captured up to the date of renal replacement therapy or transplant and censored thereafter; patients with incomplete laboratory data for assessment of treatment response were excluded. Baseline SCr was defined at day 1 of therapy.

### Outcomes

The primary study end point was HRS reversal status defined as achieving SCr ≤1.5 mg/dL at least once at any point within the treatment window. Secondary outcomes included treatment response and absolute and relative changes in SCr at day 14 (or day of treatment discontinuation). Treatment response was defined as follows: complete response, decrease in SCr from elevated baseline to a final level of ≤1.5 mg/dL at last treatment day; partial response, ≥30% decrease in SCr from elevated baseline to a final level >1.5 mg/dL at last treatment day; and no response, <30% decrease in SCr from elevated baseline at last treatment day. Overall survival and transplant-free survival up to 90 days posttreatment were also assessed. For transplant-free survival, undergoing a liver transplant or death within 90 days after start of treatment comprised the definition of event. Survival end points were also stratified by achievement of HRS reversal.

### Statistical analysis

Population adjustment methods implemented through covariate balancing propensity scoring (CBPS) were applied to balance baseline characteristics between the 2 data sources (13). Using the weights from CBPS, terlipressin-treated patients were statistically reweighted to have balanced baseline characteristics compared with those of the MO cohort as reference.

Baseline characteristics showing association with response in univariate association analyses and/or of high clinical interest were considered for inclusion in the CBPS model. All tests were 2-tailed. The final list of variables for baseline adjustment was decided based on the availability of common variables between the trial and chart review data and included age, baseline SCr, MELD, ACLF grade, encephalopathy grade, and bilirubin.

Unadjusted and adjusted outcomes were compared between terlipressin and comparator cohorts. Weighted Cox proportional hazards models and weighted Kaplan-Meier curves were used for overall and transplant-free survival comparisons. To test any residual bias resulting from the CBPS adjustment method selected, additional sensitivity analyses for terlipressin vs MO were conducted using exact matching and are contained within the Supplemental Materials (see Supplementary Digital Content 1, http://links.lww.com/CTG/B12). All data analyses were conducted in SAS 9.4 and R version 4.1.2.

## RESULTS

### Study cohorts

**Figure 1.**
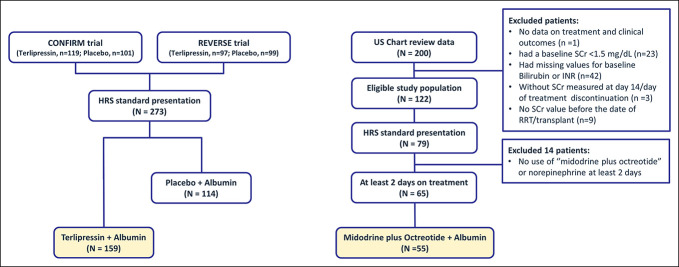
Patient attrition flow. HRS standard presentation defined by including those with SCr <5 mg/dL and baseline ACLF grades 0–2 and excluding those with MELD scores≥35 if listed for transplant. ACLF, acute-on-chronic liver failure; HRS, hepatorenal syndrome; MELD, model for end-stage liver disease; RRT, renal replacement therapy; SCr, serum creatinine.

A total of 159 terlipressin-treated patients from the pooled CONFIRM and REVERSE trials were identified as meeting inclusion criteria (i.e., including those with serum creatine <5 mg/dL and baseline ACLF grades 0–2 and excluding those with MELD scores≥35 if listed for transplant). Among 200 patients from the chart review, 65 (32.5%) of them met the initial inclusion/exclusion criteria and received at least 2 days of therapy. Our final chart review cohort included 55 patients who were treated with MO plus albumin (Figure 1). All treatment cohorts received concomitant albumin. Among the terlipressin cohort, 72 (45.3%) had previously received MO therapy. The dose for terlipressin was consistent with that used in the CONFIRM/REVERSE trials (e.g., every 5–6 hours with the dose of 1 mg and increased to 1.7 mg, if needed), while the average daily midodrine dose was 30 mg (minimal dose of 7.5 mg and maximum of 90 mg). Dose information on octreotide was not available in the US chart review.

### Baseline characteristics after adjustment

Adjusting the terlipressin cohort to MO led to a decrease in baseline median SCr (from 3.09 to 2.58 mg/dL). After reweighting of the terlipressin cohort, the effective sample size for the terlipressin cohort was n = 89 for its comparison with MO; patient cohorts were well balanced for important prognostic factors (Table 1).

**Table 1.** Comparison of baseline characteristics: terlipressin vs midodrine plus octreotide

Variable description	Statistic or category	CBPS adjusted			Unadjusted		
		Midodrine and octreotide (N = 55)	Terlipressin (N = 89)^a^	*P* value	Midodrine and octreotide (N = 55)	Terlipressin (N = 159)	*P* value
Age	Mean (SD)	58.04 (11.40)	58.09 (13.31)	0.976	58.04 (11.40)	56.22 (10.25)	0.296
	Median (Q1–Q3)	61.13 (49.50–66.04)	60.32 (51.82–65.75)		61.13 (49.50–66.04)	57.44 (50.00–64.26)	
	Range	30.00–76.00	28.11–77.96		30.00–76.00	28.11–77.96	
Sex, %	Female	41.82	45.89	0.633	41.82	42.77	0.902
	Male	58.18	54.11		58.18	57.23	
Race, %	American Indian or Alaskan Native	0.00	0.44	<0.001	0.00	1.27	0.002
	Asian	7.27	1.12		7.27	1.27	
	Black or African American	10.91	3.34		10.91	4.46	
	Hispanic or Latino	12.73	14.34		12.73	12.10	
	White	63.64	80.76		63.64	80.89	
	Unknown	5.45	0.00		5.45	0.00	
Listed for transplant at baseline, %	Yes	9.09	25.93	0.013	9.09	25.16	0.012
Baseline SCr, mg/dL	Mean (SD)	2.68 (0.79)	2.73 (0.57)	0.631	2.68 (0.79)	3.19 (0.67)	<0.001
	Median (Q1–Q3)	2.50 (2.01–3.21)	2.58 (2.33–2.90)		2.50 (2.01–3.21)	3.09 (2.63–3.62)	
	Range	1.66–4.94	1.90–4.90		1.66–4.94	1.90–4.90	
Baseline SCr (categorical), %	<3.0 mg/dL	69.09	75.60	0.339	69.09	44.65	0.002
	≥3.0–<5.0 mg/dL	30.91	24.40		30.91	55.35	
Baseline MELD score	Mean (SD)	29.93 (5.52)	29.74 (6.97)	0.845	29.93 (5.52)	30.21 (6.01)	0.744
	Median (Q1–Q3)	30.24 (25.99–34.22)	29.34 (25.08–33.16)		30.24 (25.99–34.22)	29.93 (24.98–34.28)	
	Range	15.96–42.25	16.00–40.00		15.96–42.25	16.00–40.00	
Baseline MELD cohort (categorical), %	<34	72.73	74.35	0.823	72.73	67.30	0.454
	≥34	27.27	25.65		27.27	32.70	
ACLF, %	0	5.45	2.59	0.672	5.45	0.63	0.047
	1	52.73	58.91		52.73	62.89	
	2	41.82	38.50		41.82	36.48	
Encephalopathy	Grade 0–1	83.64	83.47	0.998	83.64	79.87	0.743
	Grade 2	14.55	14.83		14.55	18.87	
	Grade 3	1.82	1.71		1.82	1.26	
Baseline bilirubin, mg/dL	Mean (SD)	9.81 (9.00)	9.23 (15.63)	0.736	9.81 (9.00)	8.33 (9.77)	0.303
	Median (Q1–Q3)	5.75 (2.58–14.55)	4.79 (2.31–10.70)		5.75 (2.58–14.55)	4.23 (2.12–9.20)	
	Range	0.30–33.90	0.30–43.70		0.30–33.90	0.30–43.70	
Baseline Child-Pugh	Class A (5–6)	1.82	1.44	0.306	1.82	2.52	0.133
	Class B (7–9)	21.82	34.90		21.82	38.36	
	Class C (10–15)	74.55	59.96		74.55	56.60	
Baseline MAP, mm Hg^b^	Mean (SD)	78.71 (12.64)	77.30 (14.24)	0.548	78.71 (12.64)	77.39 (12.48)	0.565
	Median (Q1–Q3)	74.75 (71.63–88.38)	77.17 (68.76–84.00)		74.75 (71.63–88.38)	77.06 (68.67–83.88)	
	Range	51.00–111.30	49.00–117.67		51.00–111.30	49.00–117.67	
Baseline INR cohort	<2.5	83.64	85.72	0.731	83.64	85.53	0.734
	≥2.5	16.36	14.28		16.36	14.47	
Total exposure of concomitant albumin, g	Mean (SD)	281.04 (316.31)	204.59 (215.36)	0.10	281.04 (316.31)	218.74 (185.49)	0.17
	Median (Q1–Q3)	181.25 (104.69–303.13)	161.01 (83.04–245.25)		181.25 (104.69–303.13)	167.19 (89.84–260.94)	
	Range	12.50–1,725.00	25.00–1,312.50		12.50–1,725.00	25.00–1,312.50	
Duration of concomitant albumin, d	Mean (SD)	5.40 (4.85)	4.33 (4.06)	0.146	5.40 (4.85)	4.51 (3.43)	0.214
	Median (Q1–Q3)	3.21 (2.06–5.63)	3.03 (1.70–4.79)		3.21 (2.06–5.63)	3.15 (1.65–5.28)	
	Range	1.00–28.00	1.00–20.00		1.00–28.00	1.00–20.00	
ICU admission before or during treatment, %	Yes	56.36	16.12	<0.001	56.36	16.98	<0.001
Total treatment duration, d	Mean (SD)	6.24 (5.04)	6.62 (4.95)	0.622	6.24 (5.04)	7.02 (4.57)	0.309
	Median (Q1–Q3)	3.83 (2.06–9.25)	4.89 (3.21–8.05)		3.83 (2.06–9.25)	5.23 (3.32–8.60)	
	Range	2.00–26.00	1.00–25.00		2.00–26.00	1.00–25.00	

ACLF, acute-on-chronic liver failure; HRS, hepatorenal syndrome; ICU, intensive care unit; INR, international normalized ratio; MAP, mean arterial pressure; MELD, model for end-stage liver disease; RRT, renal replacement therapy; SCr, serum creatinine.

a N = 89 is the effective sample size for the terlipressin cohort after CBPS reweighting.

b Baseline MAP for the midodrine and octreotide cohort was evaluated based on 67.3% (37/55) patients with nonmissing value in the chart review data.

### Study outcomes

After adjusting for baseline characteristics, weighted HRS reversal was observed in 52.35% of terlipressin-treated patients compared with 20% of MO-treated patients (weighted mean difference 32.35%, 95% confidence interval [CI] 17.40–47.30) (Table 2). The weighted complete response rate was 48.19% and 14.55% for the terlipressin cohort and MO cohort, respectively (weighted mean difference 33.65%, 95% CI 19.52–47.77). While 54.55% of patients had complete or partial response in the terlipressin cohort, 23.64% of patients had either complete or partial response in the MO cohort (weighted mean difference 30.91%, 95% CI 15.48%–46.34%). The change in kidney function from elevated baseline SCr and percent improvement markedly favored the terlipressin-treated cohort (Table 2). When stratifying analysis by baseline SCr value, better treatment outcomes were observed in the terlipressin cohort among patients with baseline SCr lower than 3.0 mg/dL, in which weighted HRS reversal and complete response were 36.11% (95% CI 17.10%–55.13%) and 38.9% (95% CI 21.11%–56.69%) higher, respectively, in the terlipressin cohort compared with those in the MO cohort (*P* < 0.001). While no significant differences were found between treatment cohorts among those with SCr ≥ 3.0 to <5.0 mg/dL mg/dL, albeit results were numerically trending in favor of terlipressin (see Supplementary Table 1, Supplementary Digital Content 1, http://links.lww.com/CTG/B12). In addition, the frequency of HRS reversal was 48.6% and 38.9% among patients in the terlipressin cohort who previously received MO vs those who did not, respectively (unadjusted data; *P* = 0.277; see Supplementary Table 2, Supplementary Digital Content 1, http://links.lww.com/CTG/B12). After adjustment, no statistically significant difference in the weighted HRS reversal was observed between terlipressin-treated patients with and without prior MO (63.0% vs 43.0%, *P* = 0.087) (see Supplementary Table 2, Supplementary Digital Content 1, http://links.lww.com/CTG/B12).

**Table 2.** Treatment response outcomes: terlipressin vs midodrine plus octreotide

Variable description	CBPS adjusted				Unadjusted			
	Midodrine and octreotide (N = 55)	Terlipressin (N = 89)^a^	Mean difference (95% CI)	*P* value	Midodrine and octreotide (N = 55)	Terlipressin (N = 159)	Mean difference (95% CI)	*P* value
HRS reversal, %	20.00	52.35	32.35 (17.40 to 47.30)	<0.0001	20.00	44.03	24.03 (10.80 to 37.25)	0.0004
Complete response, %	14.55	48.19	33.65 (19.52 to 47.77)	<0.0001	14.55	40.88	26.34 (14.16 to 38.51)	<0.0001
Complete or partial response, %	23.64	54.55	30.91 (15.48 to 46.34)	0.0001	23.64	53.46	29.82 (16.04 to 43.61)	<0.0001
Change in renal function (SCr) from baseline, mg/dL, mean (SD)	0.22 (1.36)	−0.70 (0.94)	−0.92 (−1.31 to −0.53)	<0.0001	0.22 (1.36)	−0.82 (1.09)	−1.04 (−1.44 to −0.64)	<0.0001
Percent improvement in renal function (SCr) from baseline, mean (SD)	−13.42 (57.70)	25.85 (35.21)	39.27 (23.04 to 55.51)	<0.0001	−13.42 (57.70)	26.14 (32.94)	39.57 (23.45 to 55.68)	<0.0001

CBPS, covariate balancing propensity scoring; CI, confidence interval; HRS, hepatorenal syndrome; SCr, serum creatinine.

a N = 89 is the effective sample size for the terlipressin cohort after CBPS reweighting.

**Figure 2.**
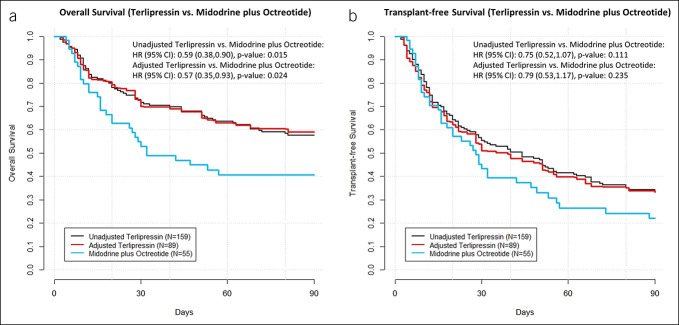
Unadjusted and CBPS-adjusted (**a**) overall survival and (**b**) transplant-free survival for terlipressin vs midodrine plus octreotide. Sample sizes for adjusted results represent effective sample size after using CBPS reweighting. CBPS, covariate balancing propensity scoring; CI, confidence interval.

**Figure 3.**
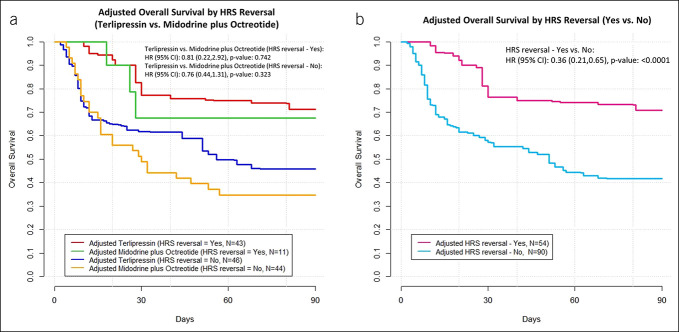
(**a**) CBPS-adjusted overall survival by HRS reversal status for terlipressin vs midodrine plus octreotide. (**b**) CBPS-adjusted overall survival by HRS reversal status regardless of treatment cohort. Sample sizes for adjusted results represent effective sample size after using CBPS reweighting. CBPS, covariate balancing propensity scoring; CI, confidence interval; HRS, hepatorenal syndrome.

**Figure 4.**
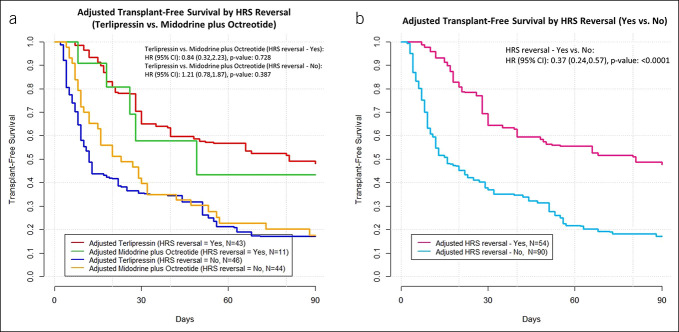
(**a**) CBPS-adjusted transplant-free survival by HRS reversal status for terlipressin vs midodrine plus octreotide. (**b**) CBPS-adjusted transplant-free survival by HRS reversal status regardless of treatment cohort. Sample sizes for adjusted results represent effective sample size after using CBPS reweighting. CBPS, covariate balancing propensity scoring; CI, confidence interval; HRS, hepatorenal syndrome.

The terlipressin cohort had increased adjusted overall survival compared with the MO cohort (adjusted hazard ratio [aHR] 0.57, 95% CI 0.35–0.93). No statistically significant difference was found for comparisons for transplant-free survival (aHR 0.79, 95% CI 0.53–1.17) (Figure 2). When stratified by achievement of HRS reversal, overall survival among patients with HRS reversal was higher than those without HRS reversal, regardless of treatment cohort (aHR 0.36, 95% CI 0.21–0.65) (Figure 3b). Similar patterns were observed for transplant-free survival (aHR 0.37, 95% CI 0.24–0.57) (Figure 4b). No difference in overall survival or transplant-free survival were found between the terlipressin and MO cohorts among patients when stratified by whether HRS reversal was achieved or not (Figures 3a and 4a). The median overall survival and transplant-free survival by treatment cohort and by HRS reversal status are summarized in Supplementary Table 3 (see Supplementary Digital Content 1, http://links.lww.com/CTG/B12). Terlipressin-treated and MO-treated patients had similar rates of liver transplantation (25.5% vs 18.2%, respectively, by day 90, *P* = 0.31), and no significant differences were observed between the 2 cohorts in the requirement of renal replacement therapy or transplantation at multiple time points (see Supplementary Table 4, Supplementary Digital Content 1, http://links.lww.com/CTG/B12).

In sensitivity analysis, an exact 1:1 matching approach was performed to examine the potential issue of residual confounding (see Supplementary Figure 1, Supplementary Digital Content 1, http://links.lww.com/CTG/B12). This sensitivity analysis confirmed HRS reversal and response results for terlipressin vs MO (see Supplementary Tables 5 and 6, Supplementary Digital Content 1, http://links.lww.com/CTG/B12). However, overall survival (HR 0.74, 95% CI 0.40–1.38, *P* = 0.35) (see Supplementary Figure 2b, Supplementary Digital Content 1, http://links.lww.com/CTG/B12) and transplant-free survival (HR 0.93, 95% CI 0.54–1.60, *P* = 0.78) were not different between terlipressin and MO (see Supplementary Figure 2d, Supplementary Digital Content 1, http://links.lww.com/CTG/B12).

Although 56.34% of patients in the MO cohort required ICU admission before or during treatment, this did not affect overall response to therapy because terlipressin was still more frequently associated with HRS reversal within the non-ICU setting (55.49% vs 16.67%, *P* < 0.05) (see Supplementary Table 7, Supplementary Digital Content 1, http://links.lww.com/CTG/B12).

## DISCUSSION

While no head-to-head clinical trials comparing terlipressin with standard of care in the United States are available, this study adds to the body of evidence demonstrating that terlipressin is associated with improved kidney function compared with MO among patients with HRS-AKI. With the use of patient-level data obtained from pooled clinical trials and a multicenter US chart review, one strength of our study was the ability to align relevant variables to evaluate outcomes by using comparable cohorts for indirect treatment comparisons. As data suggest that patients with SCr <5, ACLF grade <3, and MELD <35 may benefit most from vasoconstrictor therapy and will compose the primary population who are eligible for terlipressin therapy in the United States, we specifically studied this selected population. HRS reversal was achieved in 52.35% of our adjusted cohort who received terlipressin. Although this response is higher than those reported in the CONFIRM and REVERSE trials, it reflects a more selective population similar to those studied in trials outside of the United States (14–17). Despite using subselected patient cohorts from the original trials and chart review data sources, our study had 91.5% and 98.2% power to detect the difference in HRS reversal in the unadjusted and adjusted terlipressin and MO comparisons, respectively.

Existing literature supports the observation in our study that terlipressin plus albumin was associated with improved kidney function and outcomes compared with MO (18–21). In addition, stratifying survival by achievement of HRS reversal further corroborates the superiority of the efficacy of terlipressin. Specifically, achieving HRS reversal correlates with increased survival regardless of the type of treatment, while terlipressin achieved higher rates of HRS reversal. This has important clinical implications because it reinforces the link between improved kidney function and survival, including its potential impact on posttransplant outcomes (22). In addition, because the terlipressin cohort in our study included patients with prior MO use, HRS reversal was also evaluated between patients with and without prior MO. Because the unadjusted and weighted HRS reversal rates were not significantly different among patients who had prior MO use vs those who did not, this suggests prior MO use did not negatively bias the terlipressin cohort. Last, the MO efficacy observed in our study are consistent with those from a recent US retrospective analysis in reporting a rate of 17.4% HRS reversal among 3,918 patients treated with MO (approximately one-third of all treated population), M or O (approximately half of all patients), or norepinephrine (a quarter of all patients) (23).

Our study is not without limitations, particularly because data were obtained from 2 different sources, which could have introduced unrecognized variation among the cohorts, including differing definitions, patient visit schedules, and frequency of outcome detection. Close to two-thirds of patients in the MO cohort had missing baseline mean arterial pressure data, which then could not be adjusted for. Although survival analysis comparing cohorts derived from randomized controlled trials and retrospective data could potentially introduce selection bias, our use of CBPS to match these cohorts on baseline characteristics was essential in maintaining a valid comparison and minimizing the potential for bias. We have attempted to the best of our ability to control for differences in important prognostic factors, but residual bias is possible; we tested the results from the main CBPS adjustment using a different matching algorithm, which largely led to similar conclusions. Some differences exist between the clinical trial and chart review cohorts regarding the etiology of liver disease and precipitating events (see Supplementary Table 8, Supplementary Digital Content 1, http://links.lww.com/CTG/B12); however, the extent to which these variables may affect response to therapy is uncertain and could not be assessed in this study. Additional variables were explored for inclusion in the CBPS model, including listing for transplantation, ICU hospitalization, precipitating factors, underlying liver diagnosis, and other comorbidities; however, these were not included based on similar frequencies between comparative cohorts or a limited impact outside of other variables included in the CBPS adjustment model. Although patients in the retrospective cohort were selected based on expert chart review, verification of fulfillment of all components of the updated ICA HRS-AKI criteria was limited due to the retrospective design. The use of a nonrandomized design in our study, however, is relevant, given the recent shift in FDA decision-making process and acceptance of real-world data and evidence (24,25).

Our indirect treatment comparison supports the observation that terlipressin plus albumin improves kidney function in HRS-AKI compared with midodrine and octreotide plus albumin. Evidence in this US-based study is consistent with prior reports and merits further investigation in a future head-to-head randomized clinical trial.

## CONFLICTS OF INTEREST

**Guarantor of the article:** Viktor V. Chirikov, PhD.

**Specific author contributions:** S.A.G., D.A.S., V.V.C., X.H., and K.J.: study concept and design. V.V.C., X.H., and K.J.: acquisition of data. S.A.G., D.A.S., V.V.C., W.-J.W., X.H., and K.J.: analysis and interpretation of data. V.V.C. and W.-J.W.: drafting of the manuscripts. S.A.G., D.A.S., V.V.C., W.-J.W., X.H., and K.J.: critical revision of the manuscript for important intellectual content. V.V.C. and W.-J.W.: statistical analysis. V.V.C., X.H., and K.J.: obtained funding. V.V.C. and X.H.: administrative, technical, or material support. V.V.C., X.H., and K.J.: study supervision.

**Financial support:** This study was funded by Mallinckrodt Pharmaceuticals.

**Potential competing interests:** S.A.G. is a speakers' bureau member for Salix Pharmaceuticals, and an advisory board member, speakers' bureau member, and consultant for Mallinckrodt Pharmaceuticals. D.A.S. has consulted for BioVie, Evive, and Mallinckrodt. V.V.C. and W.-J.W. are employees of OPEN Health, which received funding to assist in the conduct of this study. X.H. and K.J. are employees of Mallinckrodt Pharmaceuticals and may own stock options.

**Data sharing statement:** Additional information and materials on the study may be provided on reasonable request to authors.

**Study Highlights**

**WHAT IS KNOWN**

✓ Evidence comparing terlipressin plus albumin and existing regimens for hepatorenal syndrome-acute kidney injury in a US population is limited.

**WHAT IS NEW HERE**

✓ Clinical trial data and real-world data were combined to compare terlipressin with midodrine and octreotide.
✓ Patients on terlipressin demonstrated improved kidney function and treatment response after adjustment for patient characteristics.
✓ Evidence in this study merits further investigation in a future head-to-head clinical trial.

## Supplementary Material
