# Supplementary material for: Terlipressin vs Midodrine Plus Octreotide for Hepatorenal Syndrome-Acute Kidney Injury: A Propensity Score–Matched Comparison
Source: Clin Transl Gastroenterol. 2023 Aug 25;14(12):e00627. doi: 10.14309/ctg.0000000000000627 (PMC10749708; doi:10.14309/ctg.0000000000000627)
Supplement: Supplementary file 1 [file ct9-14-e00627-s001.docx]

**Supplemental Digital Content (SDC)**

**Supplemental Table 1. Treatment response outcomes by baseline serum creatinine after CBPS – terlipressin vs. midodrine plus octreotide**

**Supplemental Table 2. Treatment response outcomes by prior midodrine and octreotide use among terlipressin group**

**Supplemental Table 3. Unadjusted and adjusted median overall survival and transplant-free survival (A) for terlipresion and midodrine plus octreotide (B) by HRS reversal status for terlipressin and midodrine plus octreotide**

**Supplemental Table 4. Receipt of renal replacement therapy and transplant during follow-up after CBPS and Exact Matching – terlipressin vs. midodrine plus octreotide**

**Supplemental Table 5. Comparison of baseline characteristics after CBPS and Exact Matching for terlipressin vs. midodrine plus octreotide**

**Supplemental Table 6. Treatment response outcomes after CBPS and Exact Matching – terlipressin vs. midodrine plus octreotide**

**Supplemental Table 7. Treatment response outcomes after CBPS and Exact Matching, stratified by ICU setting – terlipressin vs. midodrine plus octreotide**

**Supplemental Table 8. Etiology of liver disease, precipitating factors, and comorbidities chararacteristics at baseline - terlipressin vs. midodrine plus octreotide**

**Supplemental Figure 1. Study population for CBPS and Exact Matching analyses (terlipressin vs. midodrine plus octreotide)**

**Supplemental Figure 2. Unadjusted and adjusted overall survival using (A) CBPS, or (B) Exact matching; unadjusted and adjusted transplant-free survival using (C) CBPS, or (D) Exact Matching for terlipressin vs. midodrine plus octreotide**

# Supplemental Materials

**Supplemental Table 1. Treatment response outcomes by baseline serum creatinine after CBPS – terlipressin vs. midodrine plus octreotide**

**For baseline serum creatinine < 3.0 mg/dL**

|  | **CBPS Adjusted** | | | | **Unadjusted** | | | |
| --- | --- | --- | --- | --- | --- | --- | --- | --- |
| **Variable Description** | **Midodrine & Octreotide**  **(N= 38)** | **Terlipressin (N= 56)** | **Mean difference (95% CI)** | **P-value** | **Midodrine & Octreotide**  **(N= 38)** | **Terlipressin (N= 71)** | **Mean difference (95% CI)** | **P-value** |
| HRS reversal, % | 23.68% | 59.80% | 36.11%  (17.10 ,55.13) | 0.0003 | 23.68% | 57.75% | 34.06%  (15.95 ,52.17) | 0.0003 |
| Complete response, % | 15.79% | 54.69% | 38.90%  (21.11 ,56.69) | <.0001 | 15.79% | 52.11% | 36.32%  (19.57 ,53.08) | <.0001 |
| Complete or partial response, % | 18.42% | 57.14% | 38.72%  (20.45 ,57.00) | <.0001 | 18.42% | 57.75% | 39.33%  (22.13 ,56.53) | <.0001 |

**For baseline serum creatinine >= 3.0 to < 5.0 mg/dL**

|  | **CBPS Adjusted** | | | | **Unadjusted** | | | |
| --- | --- | --- | --- | --- | --- | --- | --- | --- |
| **Variable Description** | **Midodrine & Octreotide**  **(N= 17)** | **Terlipressin (N= 55)** | **Mean difference (95% CI)** | **P-value** | **Midodrine & Octreotide**  **(N= 17)** | **Terlipressin (N= 88)** | **Mean difference (95% CI)** | **P-value** |
| HRS reversal, % | 11.76% | 29.29% | 17.52%  (-1.65 ,36.69) | 0.0728 | 11.76% | 32.95% | 21.19%  (2.60 ,39.78) | 0.0259 |
| Complete response, % | 11.76% | 28.06% | 16.29%  (-2.77 ,35.35) | 0.093 | 11.76% | 31.82% | 20.05%  (1.52 ,38.59) | 0.0343 |
| Complete or partial response, % | 35.29% | 46.50% | 11.20%  (-15.55 ,37.96) | 0.4083 | 35.29% | 50.00% | 14.71%  (-10.8 ,40.25) | 0.2562 |

**Supplemental Table 2. Treatment response outcomes by prior midodrine and octreotide use among terlipressin cohort**

|  | **CBPS adjusted** | | | **Unadjusted** | | |
| --- | --- | --- | --- | --- | --- | --- |
|  | **Prior Midodrine and Octreotide use – Yes**  **(n= 41)** | **Prior Midodrine and Octreotide use – No**  **(n= 29)** | **P-value** | **Prior Midodrine and Octreotide use – Yes**  **(n= 72)** | **Prior Midodrine and Octreotide use – No**  **(n= 54)** | **P-value** |
| HRS reversal, % | 63.0% | 43.0% | 0.087 | 35 (48.6%) | 21 (38.9%) | 0.277 |
| Complete response, % | 56.0 % | 41.2% | 0.218 | 32 (44.4%) | 20 (37.0%) | 0.403 |
| Complete or partial response, % | 59.2% | 47.1% | 0.317 | 39 (54.2%) | 27 (50.0%) | 0.643 |

**Supplemental Table 3. Unadjusted and adjusted median overall survival and transplant-free survival (A) for terlipressin and midodrine plus octreotide (B) by HRS reversal status for terlipressin and midodrine plus octreotide**

(A)

| Treatment groups | Median overall survival  in days (95% CI) | Median transplant-free survival  in days (95% CI) |
| --- | --- | --- |
| Unadjusted terlipressin | NR (NR, NR) | 44 (29, 53) |
| Adjusted terlipressin | NR (81, NR) | 39 (22, 66) |
| Midodrine plus octreotide | 32 (26, NR) | 28 (18, 49) |

*NR: not reached

(B)

| Treatment groups | Median overall survival  in days (95% CI) | | Median transplant-free survival  in days (95% CI) | |
| --- | --- | --- | --- | --- |
|  | HRS reversal - Yes | HRS reversal - No | HRS reversal - Yes | HRS reversal - No |
| Unadjusted terlipressin | NR (NR, NR) | 63 (44, NR) | 90 (52, NR) | 22 (13, 44) |
| Adjusted terlipressin | NR (NR, NR) | 56 (25, NR) | 81 (40, NR) | 12 (9, 44) |
| Midodrine plus octreotide | NR (28, NR) | 30 (16, NR) | 49 (26, NR) | 23 (16, 47) |

*NR: not reached

**Supplemental Table 4. Receipt of renal replacement therapy and transplant during follow-up after CBPS and Exact Matching – terlipressin vs. midodrine plus octreotide**

|  | | **CBPS adjusted** | | | | **Exact matching** | | | | **Unadjusted result** | | | | |
| --- | --- | --- | --- | --- | --- | --- | --- | --- | --- | --- | --- | --- | --- | --- |
| **Variable Description** |  | **Midodrine**  **& Octreotide (N= 55)** | **Terlipressin**  **(N= 89)** | **Mean difference (95% CI)** | **P-value** | **Midodrine**  **& Octreotide**  **(N = 36)** | **Terlipressin**  **(N = 36)** | **Mean difference (95% CI)** | **P-value** | **Midodrine**  **& Octreotide (N= 55)** | **Terlipressin**  **(N= 159)** | **Mean difference (95% CI)** | **P-value** |  |
| **Transplant by Day 14 Flag, N (%)** |  | **7.27%** | **11.80%** | **0.04531**  **(-0.05656 ,0.1472)** | **0.3816** | **3 (8.33%)** | **4 (11.11%)** | **0.02778**  **(-0.1133 ,0.1688)** | **0.6957** | **4 (7.27%)** | **17 (10.69%)** | **0.03419**  **(-0.05045 ,0.1188)** | **0.4268** |  |
| **Transplant by Day 30 Flag, N (%)** |  | **10.91%** | **18.92%** | **0.08008**  **(-0.04162 ,0.2018)** | **0.196** | **3 (8.33%)** | **5 (13.89%)** | **0.05556**  **(-0.09365 ,0.2048)** | **0.4603** | **6 (10.91%)** | **26 (16.35%)** | **0.05443**  **(-0.04708 ,0.1559)** | **0.2917** |  |
| **Transplant by Day 60 Flag, N (%)** |  | **14.55%** | **23.13%** | **0.08583**  **(-0.04568 ,0.2173)** | **0.1997** | **4 (11.11%)** | **6 (16.67%)** | **0.05556**  **(-0.1087 ,0.2199)** | **0.5024** | **8 (14.55%)** | **35 (22.01%)** | **0.07467**  **(-0.03978 ,0.1891)** | **0.1998** |  |
| **Transplant by Day 90 Flag, N (%)** |  | **18.18%** | **25.45%** | **0.07273**  **(-0.06732 ,0.2128)** | **0.3072** | **5 (13.89%)** | **7 (19.44%)** | **0.05556**  **(-0.1216 ,0.2327)** | **0.5337** | **10 (18.18%)** | **38 (23.90%)** | **0.05718**  **(-0.06569 ,0.18)** | **0.36** |  |
| **RRT through End of Trt Flag (by Day 14), N (%)** |  | **14.55%** | **9.93%** | **-0.04619**  **(-0.1574 ,0.06501)** | **0.4138** | **4 (11.11%)** | **5 (13.89%)** | **0.02778**  **(-0.1297 ,0.1853)** | **0.7261** | **8 (14.55%)** | **14 (8.81%)** | **-0.0574**  **(-0.1615 ,0.04673)** | **0.2785** |  |
| **RRT by Day 30, N (%)** |  | **18.18%** | **25.02%** | **0.06843**  **(-0.06882 ,0.2057)** | **0.3268** | **5 (13.89%)** | **9 (25.00%)** | **0.1111**  **(-0.07566 ,0.2979)** | **0.2395** | **10 (18.18%)** | **38 (23.90%)** | **0.05718**  **(-0.06569 ,0.18)** | **0.36** |  |
| **RRT by Day 60, N (%)** |  | **21.82%** | **26.41%** | **0.04594**  **(-0.09768 ,0.1896)** | **0.529** | **6 (16.67%)** | **9 (25.00%)** | **0.08333**  **(-0.1092 ,0.2759)** | **0.3911** | **12 (21.82%)** | **41 (25.79%)** | **0.03968**  **(-0.09026 ,0.1696)** | **0.5479** |  |
| **RRT by Day 90g, N (%)** |  | **23.64%** | **28.06%** | **0.0442**  **(-0.1027 ,0.1911)** | **0.5537** | **7 (19.44%)** | **11 (30.56%)** | **0.1111**  **(-0.09358 ,0.3158)** | **0.2827** | **13 (23.64%)** | **44 (27.67%)** | **0.04037**  **(-0.09308 ,0.1738)** | **0.5516** |  |

** RRT: renal replacement therapy*

**Supplemental Table 5. Comparison of baseline characteristics after CBPS and Exact Matching for terlipressin vs. midodrine plus octreotide**

|  | | **CBPS Adjusted** | | | **Exact matching** ^b^ | | | **Unadjusted** | | |
| --- | --- | --- | --- | --- | --- | --- | --- | --- | --- | --- |
| **Variable Description** | **Statistic or Category** | **Midodrine & Octreotide**  **(N= 55)** | **Terlipressin**  **(N= 89)** ^a^ | **P value** | **Midodrine & Octreotide**  **(N= 36)** | **Terlipressin**  **(N= 36)** | **P value** | **Midodrine & Octreotide**  **(N= 55)** | **Terlipressin**  **(N= 159)** | **P value** |
| Age | Mean (SD) | 58.04 (11.40) | 58.09 (13.31) | 0.976 | 58.81 (11.17) | 58.74 (11.45) | 0.937 | 58.04 (11.40) | 56.22 (10.25) | 0.296 |
|  | Median (Q1 to Q3) | 61.13 (49.50 to 66.04) | 60.32 (51.82 to 65.75) |  | 62 (51.00 to 67.00) | 63.04 (53.80 to 66.07) |  | 61.13 (49.50 to 66.04) | 57.44 (50.00 to 64.26) |  |
|  | Range | 30.00 to 76.00 | 28.11 to 77.96 |  | 30.00 to 76.00 | 28.96 to 73.46 |  | 30.00 to 76.00 | 28.11 to 77.96 |  |
| Pooled Age Group, % | < 65 | 65.45% | 70.65% | 0.525 | 66.67% | 66.67% | >0.999 | 65.45% | 78.62% | 0.051 |
|  | >= 65 | 34.55% | 29.35% |  | 33.33% | 33.33% |  | 34.55% | 21.38% |  |
| Sex, % | F | 41.82% | 45.89% | 0.633 | 41.67% | 41.67% | >0.999 | 41.82% | 42.77% | 0.902 |
|  | M | 58.18% | 54.11% |  | 58.33% | 58.33% |  | 58.18% | 57.23% |  |
| Race, % | AMERICAN INDIAN OR ALASKAN NATIVE | 0.00% | 0.44% | <0.001 | 0.00% | 0.00% | 0.612 | 0.00% | 1.27% | 0.002 |
|  | ASIAN | 7.27% | 1.12% |  | 2.78% | 2.86% |  | 7.27% | 1.27% |  |
|  | BLACK OR AFRICAN AMERICAN | 10.91% | 3.34% |  | 8.33% | 5.71% |  | 10.91% | 4.46% |  |
|  | HISPANIC OR LATINO | 12.73% | 14.34% |  | 8.33% | 14.29% |  | 12.73% | 12.10% |  |
|  | WHITE | 63.64% | 80.76% |  | 75.00% | 77.14% |  | 63.64% | 80.89% |  |
|  | Unknown | 5.45% | 0.00% |  | 5.56% | 0.00% |  | 5.45% | 0.00% |  |
| White, % | Non-white | 36.36% | 19.57% | 0.031 | 25.00% | 25.00% | >0.999 | 36.36% | 20.13% | 0.016 |
|  | White | 63.64% | 80.43% |  | 75.00% | 75.00% |  | 63.64% | 79.87% |  |
| On Baseline Transplant List, % | Yes | 9.09% | 25.93% | 0.013 | 8.33% | 19.44% | 0.307 | 9.09% | 25.16% | 0.012 |
| Baseline SCr (mg/dl) | Mean (SD) | 2.68 (0.79) | 2.73 (0.57) | 0.631 | 2.91 (0.82) | 2.94 (0.62) | 0.689 | 2.68 (0.79) | 3.19 (0.67) | <0.001 |
|  | Median (Q1 to Q3) | 2.50 (2.01 to 3.21) | 2.58 (2.33 to 2.90) |  | 2.83 (2.29 to 3.31) | 2.70 (2.49 to 3.45) |  | 2.50 (2.01 to 3.21) | 3.09 (2.63 to 3.62) |  |
|  | Range | 1.66 to 4.94 | 1.90 to 4.90 |  | 1.70 to 4.94 | 1.90 to 4.20 |  | 1.66 to 4.94 | 1.90 to 4.90 |  |
| Baseline SCr Group | <3.0 mg/dl | 69.09% | 75.60% | 0.339 | 55.56% | 61.11% | 0.633 | 69.09% | 44.65% | 0.002 |
|  | >=3.0 to <5.0 mg/dl | 30.91% | 24.40% |  | 44.44% | 38.89% |  | 30.91% | 55.35% |  |
| Baseline Meld Score | Mean (SD) | 29.93 (5.52) | 29.74 (6.97) | 0.845 | 29.41 (5.94) | 29.72 (6.23) | 0.83 | 29.93 (5.52) | 30.21 (6.01) | 0.744 |
|  | Median (Q1 to Q3) | 30.24 (25.99 to 34.22) | 29.34 (25.08 to 33.16) |  | 28.76 (25.71 to 33.52) | 30 (25.50 to 33.50) |  | 30.24 (25.99 to 34.22) | 29.93 (24.98 to 34.28) |  |
|  | Range | 15.96 to 42.25 | 16.00 to 40.00 |  | 15.96 to 42.25 | 18.00 to 40.00 |  | 15.96 to 42.25 | 16.00 to 40.00 |  |
| Baseline MELD Group, % | < 34 | 72.73% | 74.35% | 0.823 | 75.00% | 75.00% | >0.999 | 72.73% | 67.30% | 0.454 |
|  | >= 34 | 27.27% | 25.65% |  | 25.00% | 25.00% |  | 27.27% | 32.70% |  |
| ACLF, % | 0 | 5.45% | 2.59% | 0.672 | 2.78% | 2.78% | >0.999 | 5.45% | 0.63% | 0.047 |
|  | 1 | 52.73% | 58.91% |  | 61.11% | 61.11% |  | 52.73% | 62.89% |  |
|  | 2 | 41.82% | 38.50% |  | 36.11% | 36.11% |  | 41.82% | 36.48% |  |
| Encephalopathy, % | Grade 0-1 | 83.64% | 83.47% | 0.998 | 80.55% | 80.55% | >0.999 | 83.64% | 79.87% | 0.743 |
|  | Grade 2 | 14.55% | 14.83% |  | 16.67% | 16.67% |  | 14.55% | 18.87% |  |
|  | Grade 3 | 1.82% | 1.71% |  | 2.78% | 2.78% |  | 1.82% | 1.26% |  |
| Baseline Bilirubin (mg/dl) | Mean (SD) | 9.81 (9.00) | 9.23 (15.63) | 0.736 | 8.73 (8.44) | 9.26 (12.02) | 0.71 | 9.81 (9.00) | 8.33 (9.77) | 0.303 |
|  | Median (Q1 to Q3) | 5.75 (2.58 to 14.55) | 4.79 (2.31 to 10.70) |  | 5.05 (2.05 to 13.30) | 4.95 (2.10 to 8.40) |  | 5.75 (2.58 to 14.55) | 4.23 (2.12 to 9.20) |  |
|  | Range | 0.30 to 33.90 | 0.30 to 43.70 |  | 0.30 to 31.90 | 0.40 to 43.70 |  | 0.30 to 33.90 | 0.30 to 43.70 |  |
| Baseline Child-Pugh, % | Class A [5-6] | 1.82% | 1.44% | 0.306 | 2.78% | 2.78% | 0.926 | 1.82% | 2.52% | 0.133 |
|  | Class B [7-9] | 21.82% | 34.90% |  | 27.78% | 30.56% |  | 21.82% | 38.36% |  |
|  | Class C [10-15] | 74.55% | 59.96% |  | 66.67% | 61.11% |  | 74.55% | 56.60% |  |
| Baseline MAP (mmhg) ^c^ | Mean (SD) | 78.71 (12.64) | 77.30 (14.24) | 0.548 | 78.80 (12.99) | 76.62 (11.48) | 0.588 | 78.71 (12.64) | 77.39 (12.48) | 0.565 |
|  | Median (Q1 to Q3) | 74.75 (71.63 to 88.38) | 77.17 (68.76 to 84.00) |  | 75.5 (73.00 to 88.00) | 75.83 (68.83 to 84.00) |  | 74.75 (71.63 to 88.38) | 77.06 (68.67 to 83.88) |  |
|  | Range | 51.00 to 111.30 | 49.00 to 117.67 |  | 51.00 to 111.30 | 52.33 to 104.67 |  | 51.00 to 111.30 | 49.00 to 117.67 |  |
| Baseline INR Group, % | < 2.5 | 83.64% | 85.72% | 0.731 | 94.44% | 88.89% | 0.674 | 83.64% | 85.53% | 0.734 |
|  | >= 2.5 | 16.36% | 14.28% |  | 5.56% | 11.11% |  | 16.36% | 14.47% |  |
| Total exposure of concomitant albumin (g) | Mean (SD) | 281.04 (316.31) | 204.59 (215.36) | 0.100 | 306.44 (365.99) | 209.93 (185.68) | 0.172 | 281.04 (316.31) | 218.74 (185.49) | 0.170 |
|  | Median (Q1 to Q3) | 181.25 (104.69 to 303.13) | 161.01 (83.04 to 245.25) |  | 200 (126.25 to 331.25) | 150 (75.00 to 300.00) |  | 181.25 (104.69 to 303.13) | 167.19 (89.84 to 260.94) |  |
|  | Range | 12.50 to 1725.00 | 25.00 to 1312.50 |  | 12.50 to 1725.00 | 25.00 to 800.00 |  | 12.50 to 1725.00 | 25.00 to 1312.50 |  |
| Duration of concomitant albumin (Days) | Mean (SD) | 5.40 (4.85) | 4.33 (4.06) | 0.146 | 5.36 (5.23) | 4.68 (3.60) | 0.634 | 5.40 (4.85) | 4.51 (3.43) | 0.214 |
|  | Median (Q1 to Q3) | 3.21 (2.06 to 5.63) | 3.03 (1.70 to 4.79) |  | 3.00 (3.00 to 5.50) | 3.00 (2.00 to 7.00) |  | 3.21 (2.06 to 5.63) | 3.15 (1.65 to 5.28) |  |
|  | Range | 1.00 to 28.00 | 1.00 to 20.00 |  | 1.00 to 28.00 | 1.00 to 14.00 |  | 1.00 to 28.00 | 1.00 to 20.00 |  |
| ICU admission before or during the treatment, % | Yes | 56.36% | 16.12% | <0.001 | 52.78% | 5.56% | <0.001 | 56.36% | 16.98% | <0.001 |
| Total Treatment Duration (Days) | Mean (SD) | 6.24 (5.04) | 6.62 (4.95) | 0.622 | 6.19 (5.30) | 6.53 (3.38) | 0.151 | 6.24 (5.04) | 7.02 (4.57) | 0.309 |
|  | Median (Q1 to Q3) | 3.83 (2.06 to 9.25) | 4.89 (3.21 to 8.05) |  | 3.50 (3.00 to 10.00) | 6.00 (4.00 to 8.50) |  | 3.83 (2.06 to 9.25) | 5.23 (3.32 to 8.60) |  |
|  | Range | 2.00 to 26.00 | 1.00 to 25.00 |  | 2.00 to 26.00 | 2.00 to 14.00 |  | 2.00 to 26.00 | 1.00 to 25.00 |  |

^a^ N = 89 is the effective sample size (ESS) for the terlipressin group after matched and reweighted based on CBPS approach

^b^ 1:1 exact matching for Midodrine & Octreotide vs. Terlipressin on age, race (white vs non-white), baseline serum creatinine, MELD, ACLF, and bilirubin

^c^ Baseline MAP for the MO group was evaluated based on 67.3% (37/55) patients with non-missing value in the chart review data

**Supplemental Table 6. Treatment response outcomes after CBPS and Exact Matching – terlipressin vs. midodrine plus octreotide**

|  | **CBPS adjusted** | | | | **Exact matching** | | | | **Unadjusted** | | | |
| --- | --- | --- | --- | --- | --- | --- | --- | --- | --- | --- | --- | --- |
| **Variable Description** | **Midodrine**  **& Octreotide (N= 55)** | **Terlipressin**  **(N= 89)** | **Mean difference (95% CI)** | **P-value** | **Midodrine**  **& Octreotide**  **(N = 36)** | **Terlipressin**  **(N = 36)** | **Mean difference (95% CI)** | **P-value** | **Midodrine**  **& Octreotide (N= 55)** | **Terlipressin**  **(N= 159)** | **Mean difference (95% CI)** | **P-value** |
| HRS reversal, % | 20.00% | 52.35% | 32.35%  (17.40 ,47.30) | <.0001 | 16.67% | 52.78% | 36.11%  (15.11 ,57.11) | 0.001 | 20.00% | 44.03% | 24.03%  (10.8 ,37.25) | 0.0004 |
| Complete response, % | 14.55% | 48.19% | 33.65%  (19.52 ,47.77) | <.0001 | 13.89% | 47.22% | 33.33%  (12.86 ,53.80) | 0.0018 | 14.55% | 40.88% | 26.34%  (14.16 ,38.51) | <.0001 |
| Complete or partial response, % | 23.64% | 54.55% | 30.91%  (15.48 ,46.34) | 0.0001 | 27.78% | 52.78% | 25.00%  (2.40 ,47.61) | 0.0307 | 23.64% | 53.46% | 29.82%  (16.04 ,43.61) | <.0001 |
| Change In Renal Function (SCr) From Baseline (mg/dl), Mean (SD) | 0.22 (1.36) | -0.70 (0.94) | -0.92  (-1.31, -0.53) | <.0001 | 0.03 (1.36) | -0.72 (0.92) | -0.75  (-1.30, -0.21) | 0.0077 | 0.22 (1.36) | -0.82 (1.09) | -1.04  (-1.44, -0.64) | <.0001 |
| Percent Improvement In Renal Function (SCr) From Baseline, Mean (SD) | -13.42 (57.70) | 25.85 (35.21) | 39.27  (23.04 ,55.51) | <.0001 | -6.89 (55.85) | 25.10 (29.35) | 31.99  (11.02 ,52.96) | 0.0033 | -13.42 (57.70) | 26.14 (32.94) | 39.57  (23.45 ,55.68) | <.0001 |

**Supplemental Table 7. Treatment response outcomes after CBPS and Exact Matching, stratified by ICU setting – terlipressin vs. midodrine plus octreotide**

**ICU**

|  | **CBPS Adjusted** | | | | **Unadjusted** | | | |
| --- | --- | --- | --- | --- | --- | --- | --- | --- |
| **Variable Description** | **Midodrine & Octreotide (N= 31)** | **Terlipressin**  **(N= 19) ^a^** | **Mean difference (95% CI)** | **P-value** | **Midodrine & Octreotide (N= 31)** | **Terlipressin**  **(N= 27)** | **Mean difference (95% CI)** | **P-value** |
| HRS reversal, % | 22.58% | 43.32% | 20.74%  (-7.62, 49.11) | 0.1486 | 22.58% | 37.04% | 14.46%  (-9.89, 38.81) | 0.2394 |
| Complete response, % | 16.13% | 34.49% | 18.36%  (-8.26, 44.97) | 0.1726 | 16.13% | 33.33% | 17.2%  (-5.67, 40.07) | 0.1375 |
| Complete or partial response, % | 25.81% | 43.23% | 17.42  (-10.83, 45.68) | 0.222 | 25.81% | 48.15% | 22.34%  (-2.97, 47.65) | 0.0825 |
| Change in renal function (SCr) from baseline (mg/dl), Mean (SD) | 0.31 (1.54) | -0.36 (1.49) | -0.67  (-1.47, 0.13) | 0.0968 | 0.31 (1.54) | -0.55 (1.26) | -0.86  (-1.60, -0.12) | 0.0234 |
| Percent improvement in renal function (SCr) from baseline, Mean (SD) | -19.30 (65.87) | 12.35 (59.63) | 31.65  (-1.35, 64.66) | 0.0598 | -19.30 (65.87) | 17.52 (41.28) | 36.82  (8.27, 65.37) | 0.0124 |

^a^ N is the effective sample size (ESS) for the terlipressin group after matched and reweighted based on CBPS approach

**Non-ICU**

|  | **CBPS Adjusted** | | | | **Unadjusted** | | | |
| --- | --- | --- | --- | --- | --- | --- | --- | --- |
| **Variable Description** | **Midodrine & Octreotide (N= 24)** | **Terlipressin**  **(N= 34) ^a^** | **Mean difference (95% CI)** | **P-value** | **Midodrine & Octreotide (N= 24)** | **Terlipressin**  **(N= 132)** | **Mean difference (95% CI)** | **P-value** |
| HRS reversal, % | 16.67% | 55.49% | 38.83%  (16.57, 61.08) | 0.0007 | 16.67% | 45.45% | 28.79%  (11.38, 46.19) | 0.0013 |
| Complete response, % | 12.50% | 52.66% | 40.16%  (18.69, 61.62) | 0.0003 | 12.50% | 42.42% | 29.92%  (14.01, 45.84) | 0.0003 |
| Complete or partial response, % | 20.83% | 56.82% | 35.99%  (12.90, 59.07) | 0.0025 | 20.83% | 54.55% | 33.71%  (15.11, 52.31) | 0.0005 |
| Change in renal function (SCr) from baseline (mg/dl), Mean (SD) | 0.11 (1.10) | -0.66 (0.87) | -0.77  (-1.23, -0.31) | 0.0013 | 0.11 (1.10) | -0.88 (1.05) | -0.98  (-1.46, -0.51) | <.0001 |
| Percent improvement in renal function (SCr) from baseline, Mean (SD) | -5.83 (45.29) | 25.90 (31.76) | 31.75  (12.92, 50.54) | 0.0011 | -5.83 (45.29) | 27.91 (30.85) | 33.73  (14.97, 52.50) | 0.0005 |

^a^ N is the effective sample size (ESS) for the terlipressin group after matched and reweighted based on CBPS approach

**Supplemental Table 8. Etiology of liver disease, precipitating factors, and comorbidities characteristics at baseline - terlipressin vs. midodrine plus octreotide**

|  | | **CBPS Adjusted** | | | **Unadjusted** | | |
| --- | --- | --- | --- | --- | --- | --- | --- |
| **Variable Description** | **Statistic or Category** | **Midodrine & Octreotide**  **(N= 55)** | **Terlipressin**  **(N= 89)^a^** | **P value** | **Midodrine & Octreotide**  **(N= 55)** | **Terlipressin**  **(N= 159)** | **P value** |
| Underlying cause of cirrhosis, (%) | Acute alcoholic cirrhosis, (%) | 40.00% | 57.46% | 0.043 | 40.00% | 61.01% | 0.007 |
|  | Hepatitis B, (%) | 7.27% | 1.25% | 0.040 | 7.27% | 1.26% | 0.020 |
|  | Hepatitis C, (%) | 16.36% | 14.88% | 0.813 | 16.36% | 18.24% | 0.754 |
|  | Primary biliary cholangitis, (%) | 1.82% | 3.34% | 0.581 | 1.82% | 3.77% | 0.482 |
|  | Non-Alcoholic Steatohepatitis (NASH), (%) | 21.82% | 72.33% | <0.001 | 21.82% | 72.96% | <0.001 |
| Precipitating events, (%) | Diuretics treatment, (%) | 40.00% | 16.38% | 0.005 | 40.00% | 13.84% | <0.001 |
|  | GI bleeding, (%) | 18.18% | 7.64% | 0.098 | 18.18% | 5.66% | 0.005 |
|  | Large-volume paracentesis, (%) | 49.09% | 12.30% | <0.001 | 49.09% | 13.21% | <0.001 |
|  | None of three precipitating events above, (%) | 27.27% | 73.21% | <0.001 | 27.27% | 75.47% | <0.001 |
| Comorbidities, (%) | Diabetes Mellitus, (%) | 25.45% | 30.85% | 0.484 | 25.45% | 28.30% | 0.684 |
|  | Chronic kidney disease, (%) | 25.45% | 4.93% | <0.001 | 25.45% | 8.18% | <0.001 |
|  | Congestive heart failure, (%) | 1.82% | 2.63% | 0.739 | 1.82% | 3.77% | 0.482 |
|  | Hypertension, (%) | 38.18% | 46.91% | 0.306 | 38.18% | 47.17% | 0.248 |
|  | Coronary artery disease, (%) | 7.27% | 5.37% | 0.598 | 7.27% | 8.81% | 0.724 |
|  | None of these, (%) | 45.45% | 37.67% | 0.360 | 45.45% | 38.99% | 0.400 |

^a^ N = 89 is the effective sample size (ESS) for the terlipressin group after CBPS reweighting

**Supplemental Figure 1. Study population for CBPS and Exact Matching analyses (terlipressin vs. midodrine plus octreotide)**


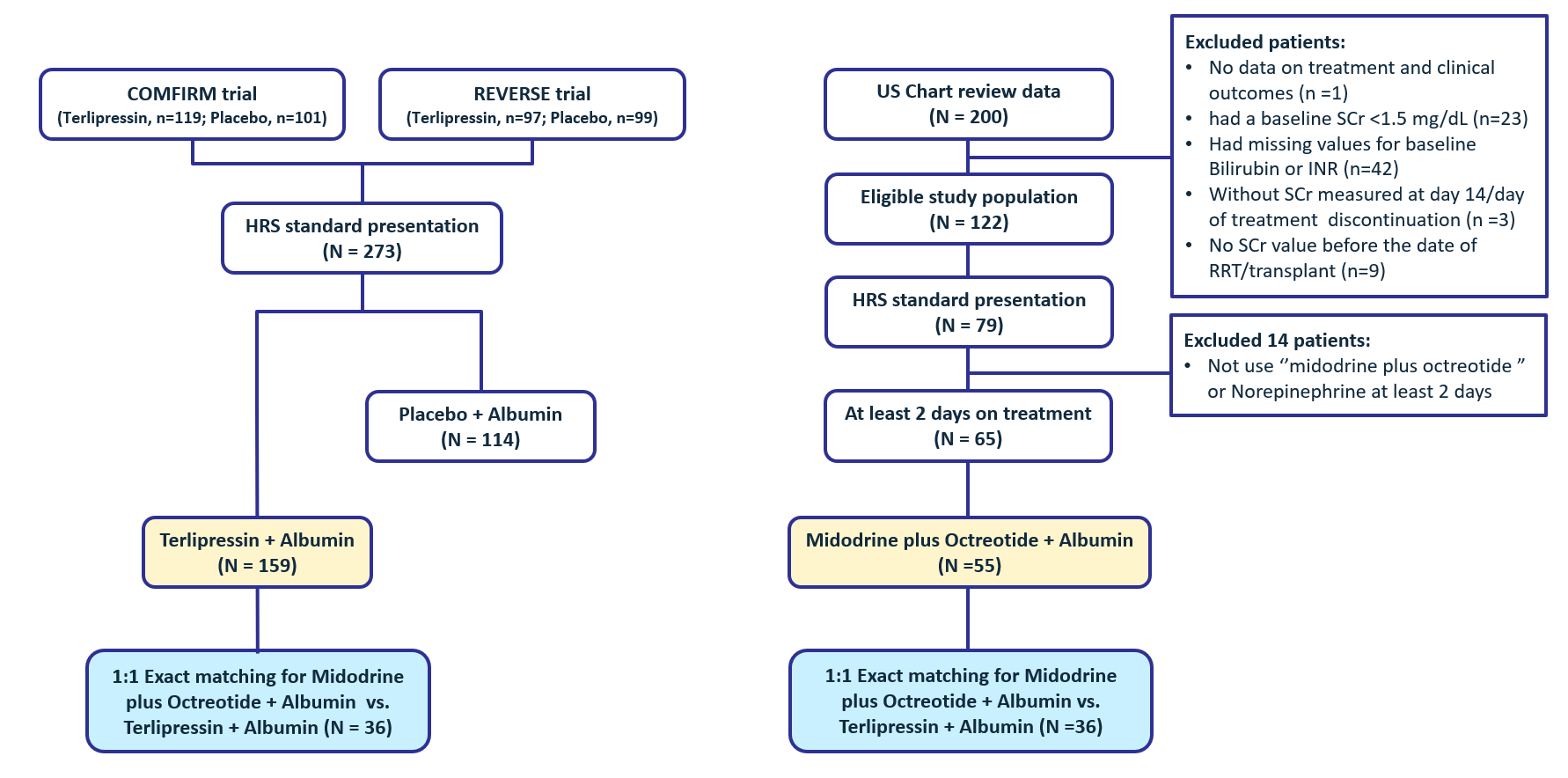


* A list of variables for exact matching:1:1 exact matching for Midodrine & Octreotide vs. Terlipressin on age, race (white vs non-white), baseline serum creatinine, MELD, ACLF, and bilirubin.

**Supplemental Figure 2. Unadjusted and adjusted overall survival using (A) CBPS, or (B) Exact matching; unadjusted and adjusted transplant-free survival using (C) CBPS, or (D) Exact Matching for terlipressin vs. midodrine plus octreotide**

| (A) | (B) |
| --- | --- |
| 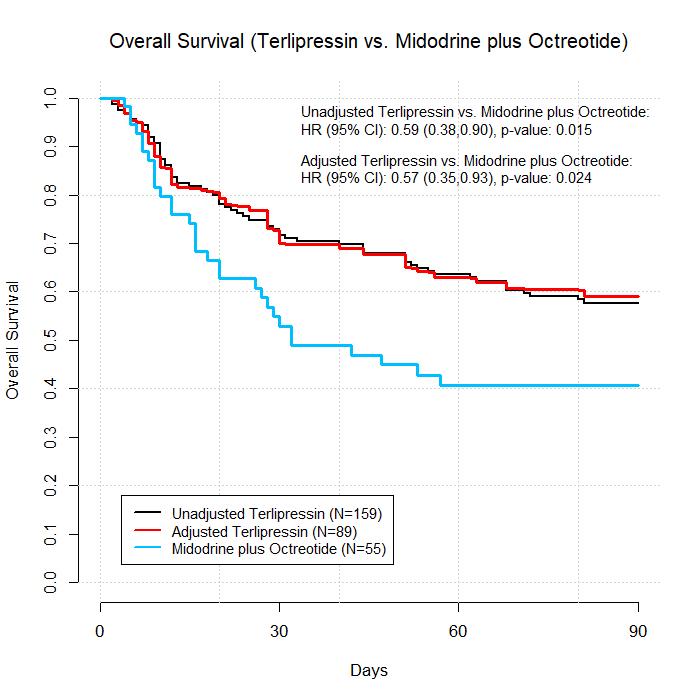 | 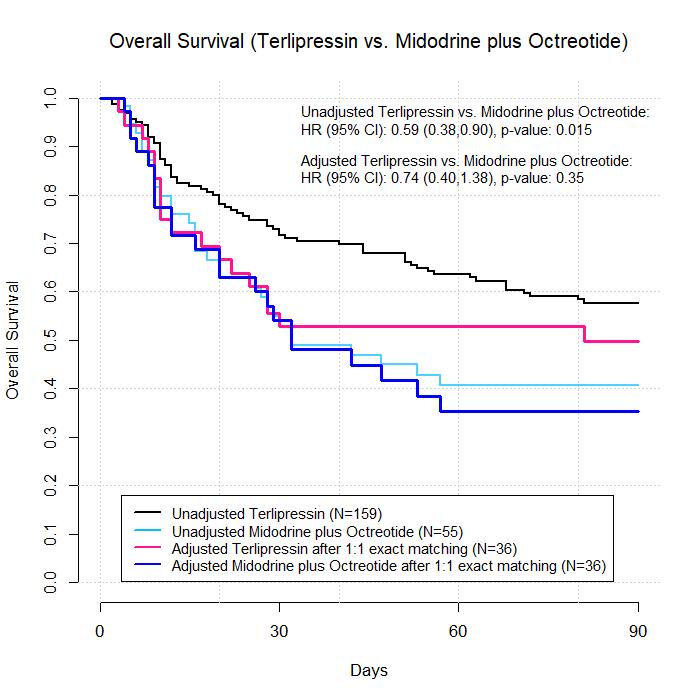 |
| (C) | (D) |
| 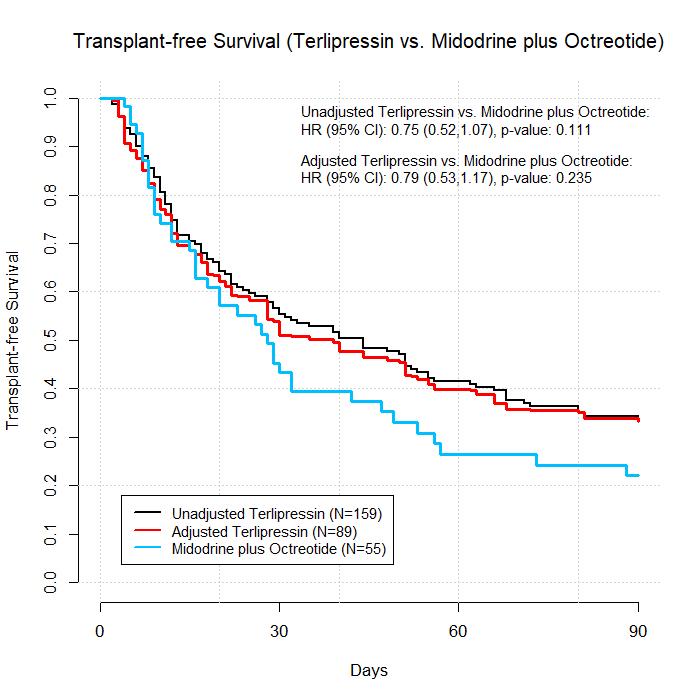 | 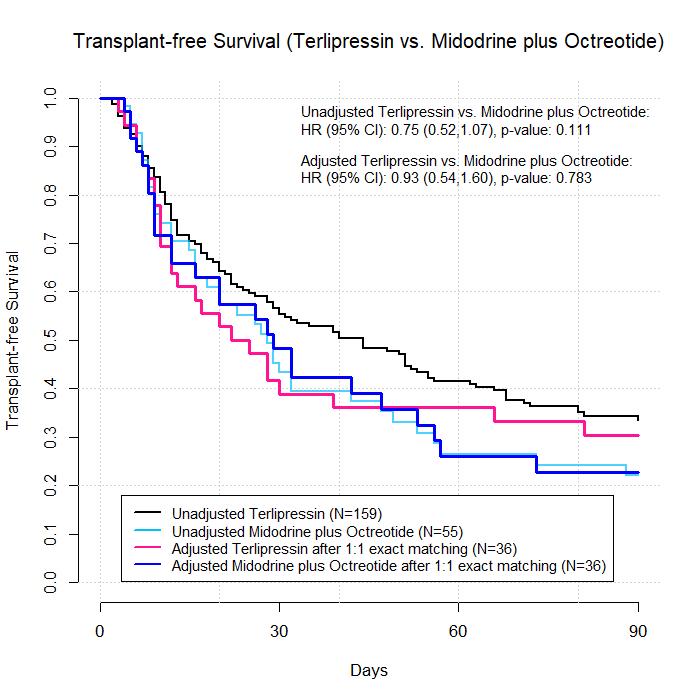 |

| ***Terlipressin vs Midodrine plus Octreotide*** | **CBPS adjusted** | | **Exact Matching** | |
| --- | --- | --- | --- | --- |
|  | HR (95% CI) | p-value | HR (95% CI) | p-value |
| Adjusted OS | 0.574 (0.355 ,0.928) | 0.024 | 0.744 (0.401 ,1.383) | 0.350 |
| Adjusted TFS | 0.787 (0.529 ,1.169) | 0.235 | 0.927 (0.538 ,1.597) | 0.783 |
